# Knock-Out Serum Replacement and Melatonin Effects on Germ Cell Differentiation in Murine Testicular Explant Cultures

**DOI:** 10.1007/s10439-017-1847-z

**Published:** 2017-05-09

**Authors:** Ahmed Reda, Halima Albalushi, Sheyla Cisneros Montalvo, Mirja Nurmio, Zeliha Sahin, Mi Hou, Niels Geijsen, Jorma Toppari, Olle Söder, Jan-Bernd Stukenborg

**Affiliations:** 1 Department of Women’s and Children’s Health, Pediatric Endocrinology Unit, Q2:08, Karolinska Institutet and Karolinska University Hospital, 17176 Stockholm, Sweden; 2 NORDFERTIL Research Lab Stockholm; Q2:08; Karolinska Institutet and Karolinska University Hospital, 17176 Stockholm, Sweden; 3 College of Medicine and Health Sciences, Sultan Qaboos University, Muscat, Oman; 4 Department of Physiology, Institute of Biomedicine, University of Turku, Turku, Finland; 5 Hubrecht Institute–KNAW and University Medical Center Utrecht, Utrecht, The Netherlands; 6 Department of Pediatrics, Turku University Hospital, Turku, Finland

**Keywords:** *In vitro* spermatogenesis, Germ cell, DDX4, CREM, KSR

## Abstract

Finding robust culture conditions for *in vitro* maturation (IVM) of male germ cells is still a challenge. Recently, a testis organ culture method, using Knockout Serum Replacement (KSR), was suggested as a promising approach. However, the efficiency of that model is still not optimal. Hence, we have tried to establish the culture conditions in two laboratories, and to improve the reliability of the culture system to generate mature germ cells. Male mice at three days of age were sacrificed. Testes were cut into small pieces which were cultured atop agarose stands, using Minimum Essential Medium alpha supplemented with different supplements; melatonin, Glutamax, and different concentrations of KSR. The results showed that the duration of culture beyond 18 days had an impact on the number of differentiated germ cells. Supplementation with melatonin and Glutamax revealed a positive influence on the efficiency of male germ cell differentiation *in vitro*. Furthermore, the results confirmed that KSR had a positive effect on germ cell maturation and testosterone production, with a concentration of at least 10%. In conclusion, this study emphasizes the beneficial role of at least 10% KSR in the IVM of germ cells.

**Electronic supplementary material:**

The online version of this article (doi:10.1007/s10439-017-1847-z) contains supplementary material, which is available to authorized users.

## Introduction

The generation of robust and reliable culture conditions for *in vitro* maturation (IVM) of male germ cells has been the topic in research for more than a century. Much in vogue during the 1960’s and 1970’s,^27^,^28^ testicular explant culture conditions have been replaced by other methodologies culturing single cell suspensions of germ cells on somatic feeder cells,^10^ or in matrices providing an artificial three-dimensional (3D) microenvironment.^29^ However, in 2011 the testicular explant approach was revived after a publication by Sato and colleagues demonstrating for the first time the production of functional sperm in explants from post-natal mice,^23^ using in principle the same methodology as described nearly 50 years ago.^34^ The main idea behind the technique is to place the cultured tissue at the interface between the gaseous phase; where it can reach oxygen, and the liquid phase; where it can reach the nutrients provided by the cell culture medium.^9^ The success of the newly-described condition is strongly connected to the supplementation of the medium with Knockout Serum Replacement (KSR) or AlbuMAX as replacement for fetal calf or fetal bovine serum.^9^,^14^,^16^

To date, the culture conditions described by Sato and colleagues in 2011 have been applied to different settings including cryopreserved murine testicular tissue,^7^,^38^ adult murine testicular tissue,^24^ single cell suspensions obtained from juvenile murine testis,^25^ juvenile rat, bovine,^12^,^16^,^20^ and pre-pubertal human testicular tissue^6^ and even spermatogonial stem cell lines reintroduced into the seminiferous tubules of immature mice and cultured afterwards under organ culture approaches as previously described.^25^ Taking all these innovative experiments together, the testis explant system has been proven to work in rodents and can be considered today as the most promising culture approach to further investigate male germ cell production and biology *in vitro*. However, despite the increasing number of reports employing this technique, the number of groups using the system successfully is relatively small. This is most probably due to the low efficiency rate of differentiation, which limits the application in many areas of interest, as, for example, toxicology tests in the pharmaceutical industry.^4^

Hence, in this study, we first tried to establish some baseline culture conditions in two independent laboratories to ensure the reliability and replicability of the methods. Afterwards, we aimed to define the value of KSR, because it was a medium component in all studies showing successful IVM of male germ cells. We also investigated the effect of Glutamax and melatonin supplementation as potential improvements to the explant method. We assessed germ cell proliferation and maturation by (1) light microscopy of transverse sections of the explants, (2) staining with markers for mature germ cells (DEAD Box Protein 4 (DDX4)/CREM) and dividing germ cells (DDX4/KI-67) and (3) by sorting cells with flow cytometry by size and ploidy, in addition to immunostaining for γH2AX-Ser139 as a marker for meiosis. We also measured testosterone (*T*) levels in the medium as a marker of Leydig cell function.

## Materials and Methods

For more detailed information, please see “supplementary Materials and methods”.

### Animals

Wild-type C57/BL6 pregnant mice were purchased from Charles River (Sulzfeld, Germany), transported with their mothers to Karolinska Institutet (Stockholm, Sweden). Pregnant C57/BL6 mice were housed at the Animal Center of the University of Turku (Turku, Finland) in a controlled environment with free access to food and water *ad libitum*.

### Ethical Approval

The use and handling of animals was approved by the ethics committee for experimental laboratory animals at Karolinska Institutet (N489/11 and N280/14) and the University of Turku, Finland.

### Testicular Tissue Culture

The testicular explant culture methods for testicular tissue, described originally by Sato *et al*.^23^ To prepare the agarose pillar, pre-autoclaved 0.7% SeaKem^®^ LE agarose (50004, Lonza, Basel, Switzerland) was mixed with the relevant culture medium 1:1 v/v to give a final concentration of 0.35% agarose. The different culture conditions used were; (1) minimum essential medium alpha (MEMα; 22561-021, Gibco) + melatonin (M5250, Sigma Aldrich, Munich, Germany, final concentration 10^−7^ M) (2) MEMα + Glutamax (32561-029, Gibco, 1.87 mM Glutamax), (3) MEMα + 10% Knockout Serum Replacement (KSR, 10828-028, Gibco), (4) MEMα + Glutamax + melatonin, (5) MEMα + Glutamax + 10% KSR, (6) MEMα + Glutamax + 10% KSR + melatonin, (7) MEMα + melatonin + 0.1% KSR, (8) MEMα + melatonin + 1% KSR, (9) MEMα + melatonin + 10% KSR, and (10) MEMα + melatonin + 20% KSR. The tissue was cultured at 34.5 °C, normal oxygen tension (21%) and 5% CO_2_.

### Embedding and Sectioning

Cultured tissue explants were collected and fixed either in Bouin’s solution (HT10132, Sigma-Aldrich) or in formaldehyde 4% in phosphate buffered saline (PBS) (02176, Histolab, Gothenburg, Sweden) at 4 °C overnight. Embedding of the samples was performed as published recently.^20^

### Periodic Acid-Schiff (PAS) Staining and Morphologic Evaluation

Sections of the samples were de-paraffinized in xylene and rehydrated in descending ethanol concentrations at RT. The PAS kit (101646, Merck, Darmstadt, Germany) was applied to stain the sections in accordance with the manufacturer’s protocol. For the morphologic evaluation, the different types of germ cells were identified based on the morphologic aspects that were described earlier.^22^

### Immunostaining

DDX4/KI-67 or DDX4/CREM double staining were performed as described previously.^19^ The sections were incubated with either rabbit polyclonal anti-DEAD Box Protein 4 primary antibody (DDX4; ab13840, Abcam, Cambridge, UK) or rabbit immunoglobulins G (IgGs; ab27478, Abcam) as a negative control overnight at 4 °C, both diluted in the blocking buffer (Tris buffered saline/normal chicken serum/bovine serum albumin or TBS/NChS/BSA). Afterwards, the sections were washed with TBS and incubated for 30 min at room temperature (RT) with Horseradish peroxidase (HRP)-conjugated chicken anti-rabbit secondary antibody (SC-2963, Santa Cruz) diluted in blocking buffer (TBS/NChS/BSA). After washing with TBS, the TSA™ Plus Fluorescein System (NEL741001KT, Perkin Elmer Life Sciences, Boston, USA) was applied in accordance with the manufacturer’s protocol.

In order to strip away the antibodies from the sections before staining with the second primary antibody, antigen retrieval was repeated. Afterwards, the sections were incubated with either rabbit monoclonal anti-KI-67 primary antibody (ab16667, Abcam), rabbit polyclonal anti-CREM primary antibody (SC-440, Santa Cruz) or rabbit IgGs as a negative control overnight at 4 °C, all diluted in the blocking buffer. The TSA™ Plus Cyanine 3 System (NEL744001KT, Perkin Elmer Life Sciences) was applied according to the manufacturer’s protocol.

Immunohistochemistry with the anti-deleted in azoospermia like protein (DAZL) antibody (rabbit polyclonal, ab-34139; Abcam) was performed as follows: antigen retrieval for sections of formaldehyde 4% fixed samples after de-paraffinization was performed in 50 mM glycine (pH 3.5; ≥90 °C maintained for 10 min) and the primary antibody was applied at 1.0 μg/mL for overnight incubation at 4 °C in 0.1% BSA/TBS. Negative control sections were incubated with 0.1% BSA/TBS lacking primary antibodies. Subsequent steps were performed at RT, with TBS washes between incubations. Primary antibody binding was detected using Vectastain ABC kit Universal according to the manufacturer’s instructions (PK-7200, Vector Laboratories).

### Quantitative Analysis of Testicular Cell Populations by Flow Cytometry

Four cultured testis pieces from each treatment were pooled and prepared for flow cytometric analysis as previously described by Rotgers and colleagues.^21^ Briefly, cultured testis pieces were cut using McPherson-Vannas scissors and enzymatically digested with 1 mg/mL collagenase/dispase (10269638001, Roche, Basel, Switzerland), 1 mg/mL hyaluronidase (H3506, Sigma–Aldrich), 1 mg/mL DNAse1 (DN-25, Sigma–Aldrich). Cell suspensions were filtered, subsequently fixed and permeabilized using 4% paraformaldehyde and 90% methanol. To assess the different germ cell populations in the testicular pieces, immunolabeling with mouse anti- γH2AX-Ser139 antibody (05-636, EMD Millipore Billerica, MA, USA), which is a marker for meiosis, was performed.

### Testosterone Assay

For the evaluation of testosterone levels, the collected media was thawed and the Enzyme Linked Immunosorbent Assay (ELISA) kit (EIA-1559, DRG instruments, Marburg, Germany) with an intra-assay CV < 5% and inter-assay CV < 10% was used according to the manufacturer’s protocol.

### Statistical Analysis

To perform the statistical analysis, *t* test, One-way ANOVA and ANOVA on ranks were applied, using the Sigma Plot software ver.12.0 (Systat Software Inc., IL, USA) as stated in the figure legends. The means and standard deviations were used in the figures as indicated and each experimental condition was repeated at least 3 times. A *p* value ≤0.05 was considered to indicate a significant difference.

## Results

### Time Dependent Effects on Germ Cells Maturation in Testicular Tissue Cultures

Testicular tissue obtained from 3 d*pp* mice was cultured for 18, 35 and 56 days using MEMα + 10% KSR as a basic culture medium. Samples were collected and fixed in paraformaldehyde and Bouin’s solution for further morphologic and immunofluorescent analysis, to reveal the effect of culture duration under these conditions.

**Figure 1:**
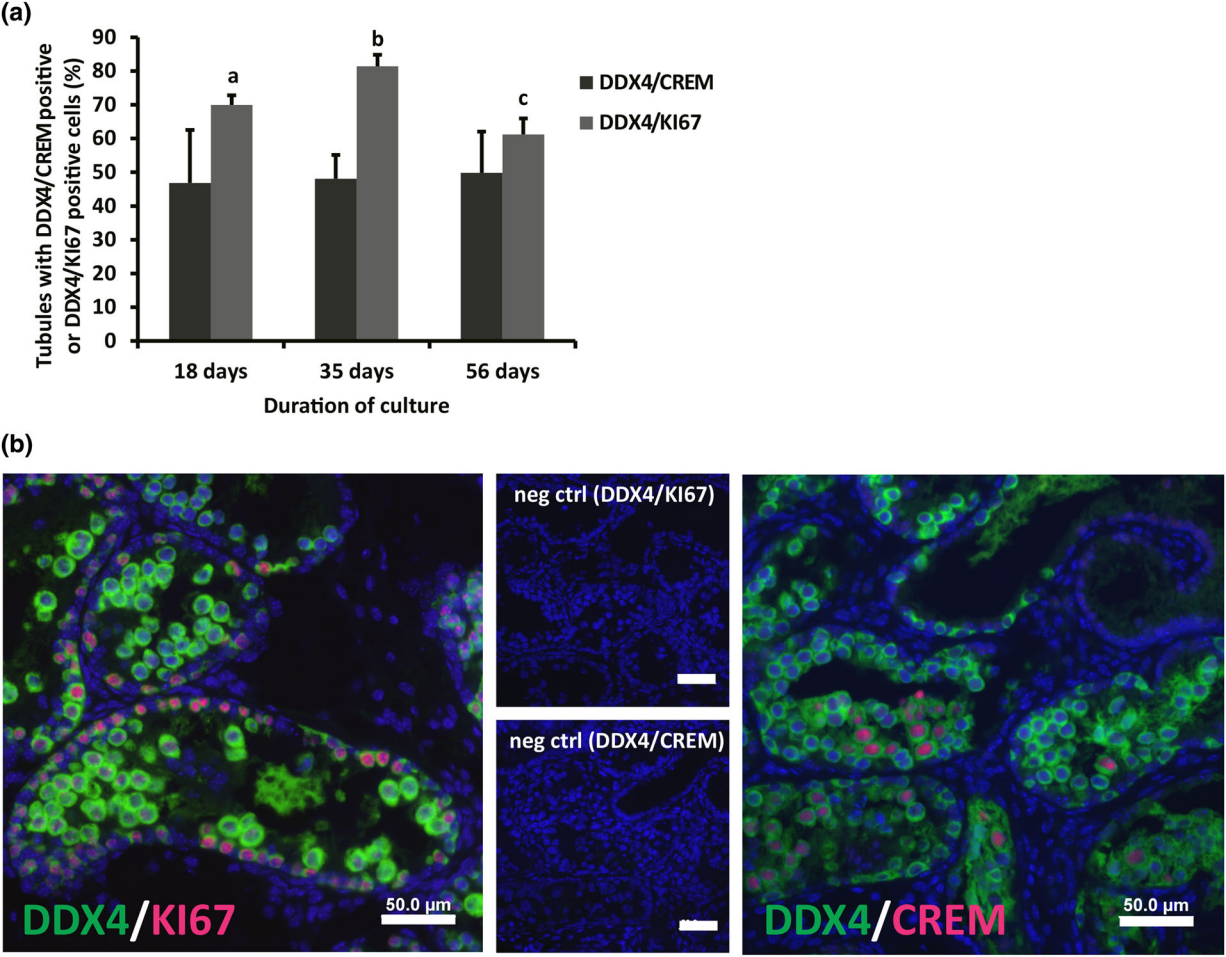
Effect of culture time on the murine pre-pubertal testicular tissue. (a) Percentage of tubules containing DDX4/CREM positive or DDX4/KI-67 positive cells after 18, 35, and 56 days of culturing 3 d*pp* mouse testicular tissue in minimum essential medium alpha (MEMα) + 10% knockout serum replacement (KSR). Three to five biological replicates were evaluated for each time point. Values are represented in mean ± standard deviation. For statistical analysis, one-way ANOVA followed by Holm-Sidak post hoc analysis was performed. Different letters represent statistical significance (*p* < 0.05 a and c vs. b; *p* < 0.001: b vs. c). (b) Double immunofluorescence staining for DDX4 (green)/KI-67 (red) and DDX4 (green)/CREM (red) of paraformaldehyde (PFA) 4% fixed sections of 3 d*pp* mouse testicular tissue cultured in MEMα + KSR 10% for 35 days. Small pictures represent the negative controls. Scale bar is 50 µm.

The highest percentage of seminiferous tubules containing proliferating germ cells, identified by DDX4/KI-67 double positive cells, was observed after 35 days in culture, when compared to 18 or 56 days of culture (81 ± 3% compared to 69 ± 2 and 61 ± 4% respectively), while the cultured tissue at 18 days showed significantly higher germ cell proliferation index compared to the tissue cultured at 56 day, as shown in Figs. 1a and 1b.

The evaluation of seminiferous tubules containing DDX4/CREM positive cells, showed no difference over the culture period (46 ± 15 to 49 ± 12%). However, when comparing the numbers of cells positive for DDX4 or DDX4/CREM in those tubules, significantly more DDX4 and DDX4/CREM expressing cells could be observed after 18 days, compared to 35 or 56 days *in vitro* (Table 1). Round spermatids could be observed in all tissue fragments analyzed 18, 35 and 56 days *in vitro*. After 35 or 56 days in culture, elongated spermatids could be observed in 67 or 60% of all tissue fragments analyzed, respectively.

**Table 1:** Average numbers of total, DDX4 positive and DDX4/CREM double positive cells per tubule cultured in different conditions (A–M).

Culture condition (MEMα based)		Culture period (days)	Most advanced germ cell type	Total cells/tubule ± SD	DDX4 pos. cells/tubule ± SD	Statistical significance (DDX4 pos. cells) (***p < 0.001; **p < 0.01; *p < 0.05)	DDX4/CREM pos. cells/tubule ± SD	Statistical significance (DDX4/CREM pos. cells) (***p < 0.001; **p < 0.01; *p < 0.05)	Percentage (%) of tissue fragments showing DDX4/CREM pos. cells
A	+10% KSR	18	rSpt	148 ± 52	67 ± 28	A > B: *** A > C: ***	22 ± 16	A > B: *** A > C: ***	100
B	+10% KSR	35	eSpt	104 ± 33	47 ± 27	B < A: *** B < I: ***	11 ± 10	B < A: *** B < I: *** B < H: *	100
C	+10% KSR	56	eSpt	110 ± 42	42 ± 17	C < A: ***	10 ± 7	C < A: ***	100
D	+0% KSR + Glx	35	Spg	93 ± 23	13 ± 5	D vs. E: NS	0 ± 0	D vs. E: NS	0
E	+0% KSR + Mel	35	Spg	78 ± 49	13 ± 7	E < I: ** E < K: ** E vs. D: NS	0 ± 0	E < I: *** E < K: *** E vs. D: NS	0
F	+0.1% KSR + Mel	35	Spg	50 ± 21	8 ± 4	F < I: ** F < K: **	0 ± 0	F < I: *** F < K: **	0
G	+1% KSR + Mel	35	Spc	100 ± 53	22 ± 14	G < I: ** G < K: **	3 ± 4	G < I: *** G < K: ***	100
H	+10% KSR + Glx	35	eSpt	116 ± 42	54 ± 27	H < I: **	19 ± 14	H < I: ** H > B: *	100
I	+10% KSR + Mel	35	eSpt	210 ± 81	81 ± 57	I > E: ** I > F: ** I > G: ** I > B: *** I > J:*** I > H:**	31 ± 28	I > E: *** I > F: *** I > G:*** I > B: *** I > J: *** I > H: **	100
J	+10% KSR + Mel + Glx	35	eSpt	129 ± 80	45 ± 33	J < I: ***	10 ± 7	J < I: ***	100
K	+20% KSR + Mel	35	eSpt	126 ± 78	41 ± 40	K > E: ** K > F: ** K > G:**	21 ± 26	K > E: *** K > F: ** K > G:***	100
L	+Glx (3 weeks) + 10% KSR (2 weeks)	35	rSpt	124 ± 23	62 ± 12	L < M: ***	14 ± 9	L < M: **	100
M	+Mel +Glx (3 weeks) + 10% KSR (2 weeks)	35	rSpt	111 ± 25	51 ± 16	M > L: ***	19 ± 9	M > L: **	100

*KSR* knockout serum replacement, *Glx* Glutamax, *Mel* melatonin, *MEMα* minimum essential medium alpha, *SD* standard deviation, *Spg* spermatogonia, *Spc* spermatocytes, *rSpt* round spermatids, *eSpt* elongated spermatids, *NS* not significantly different

The presence of DAZL positive germ cells (differentiating spermatogonia and early spermatocytes) was confirmed by immunohistochemistry over the whole culture period, suggesting a viable spermatogonia population (Supplementary Fig. 1).

### Effect of Glutamax and Melatonin

Testicular tissue obtained from 3 d*pp* mice was cultured for 35 days using MEMα + 10% KSR as a basic culture medium. To assess the effect of melatonin, which has been observed in a previous study when using testicular tissue of CD-1 mice,^4^ the basic culture medium used in the first experiments was supplemented with either melatonin, Glutamax, or a combination of both. Samples collected after 35 days of culture were evaluated for the percentage of tubules showing an expression of DDX4/KI-67, DDX4/CREM, as well as for key morphologic features of specific germ cell subtypes.

**Figure 2:**
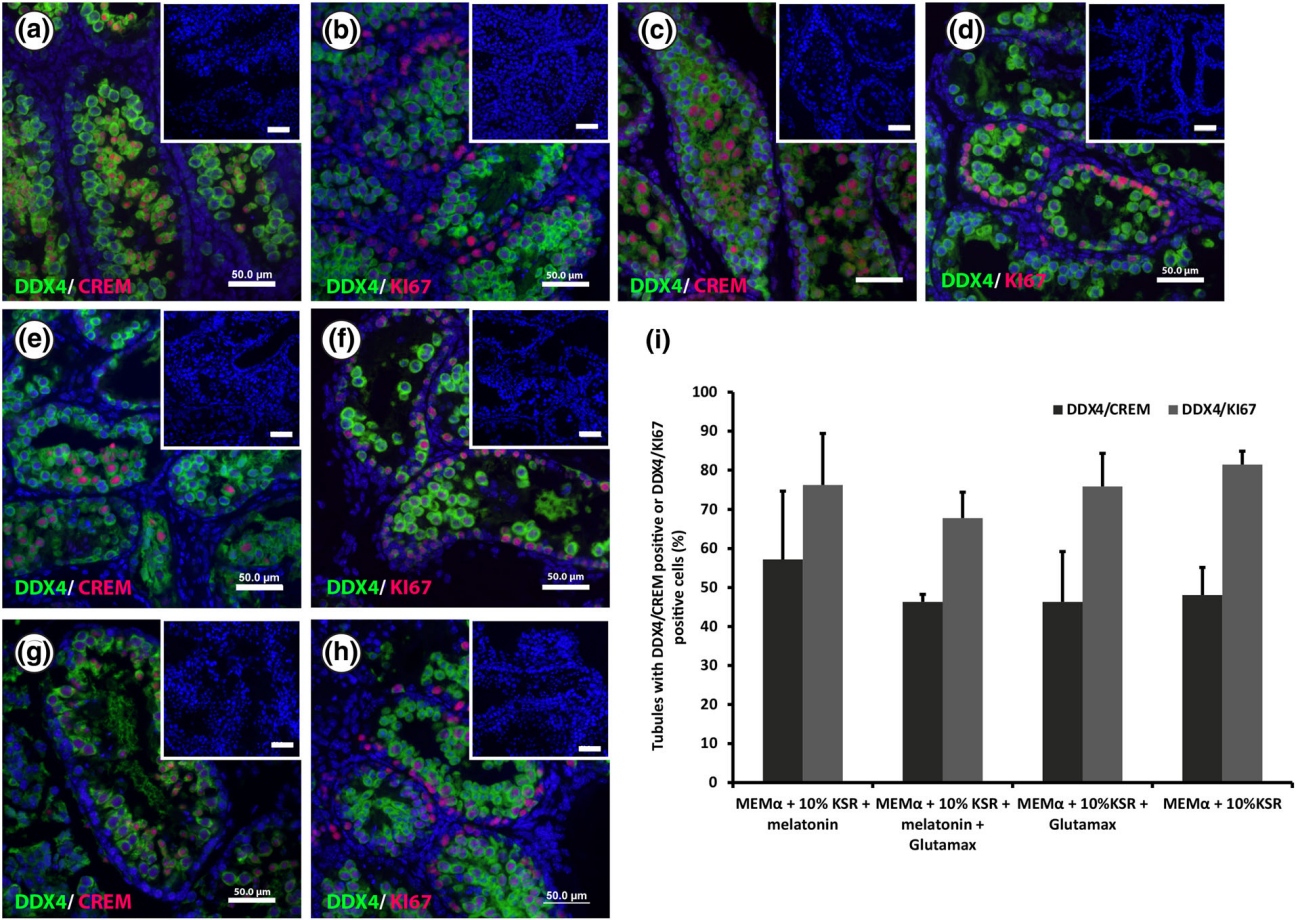
Effect of melatonin and Glutamax on murine pre-pubertal testicular tissue cultured *in vitro*. (a–h) Double immunofluorescence staining for DDX4 (green)/KI-67 (red) and DDX4 (green/CREM (red) of 4% paraformaldehyde (PFA) fixed sections of 3 d*pp* mouse testicular tissue cultured in minimum essential medium alpha (MEMα) + knockout serum replacement (KSR) 10% + melatonin for 35 days (a, b), MEMα + KSR 10% + melatonin + Glutamax (c, d), MEMα + KSR 10% + Glutamax (e, f), and MEMα + KSR 10% (g, h). Small pictures in the upper right corners represent the negative controls. Scale bar is 50 µm. (i) Percentage of tubules containing DDX4/CREM positive or DDX4/KI-67 positive cells after 35 days of culturing 3 d*pp* mouse testicular tissue in MEMα + 10% KSR + melatonin, MEMα + 10% KSR + melatonin + Glutamax, MEMα + 10% KSR + Glutamax, or MEMα + 10% KSR. Three to five biological replicates were evaluated for each time point. Values are represented in mean ± standard deviation. For statistical analysis, one-way ANOVA was performed. However, no significant differences were observed between the groups.

The results revealed that supplementing the culture medium with melatonin, Glutamax, or a combination of both had no significant effect on the percentages of tubules containing DDX4/CREM double positive cells. No significant difference could be measured when comparing the four conditions (46 ± 1 to 57 ± 17%) as shown in Figs. 2a, 2c, 2e, 2g, and 2i. However, further evaluations of DDX4 and DDX4/CREM expressing cells in those tubules revealed a significantly higher number of cells expressing DDX4 and DDX4/CREM in culture conditions containing 10% KSR and melatonin compared to the other conditions (Table 1). In addition, more DDX4/CREM positive cells could be observed in culture conditions using 10% KSR with Glutamax compared to conditions with only 10% KSR as supplement (19 ± 14 vs. 11 ± 10, respectively) (Table 1).

To investigate the proliferation activity of germ cells cultured in these conditions, percentages of seminiferous tubules containing double positive cells for DDX4 and KI-67 were compared to the total number of tubules. The results showed that there was no significant difference between the different groups (67 ± 6 to 81 ± 3%), as shown in Figs. 2b, 2d, 2f, 2h, and 2i.

**Figure 3:**
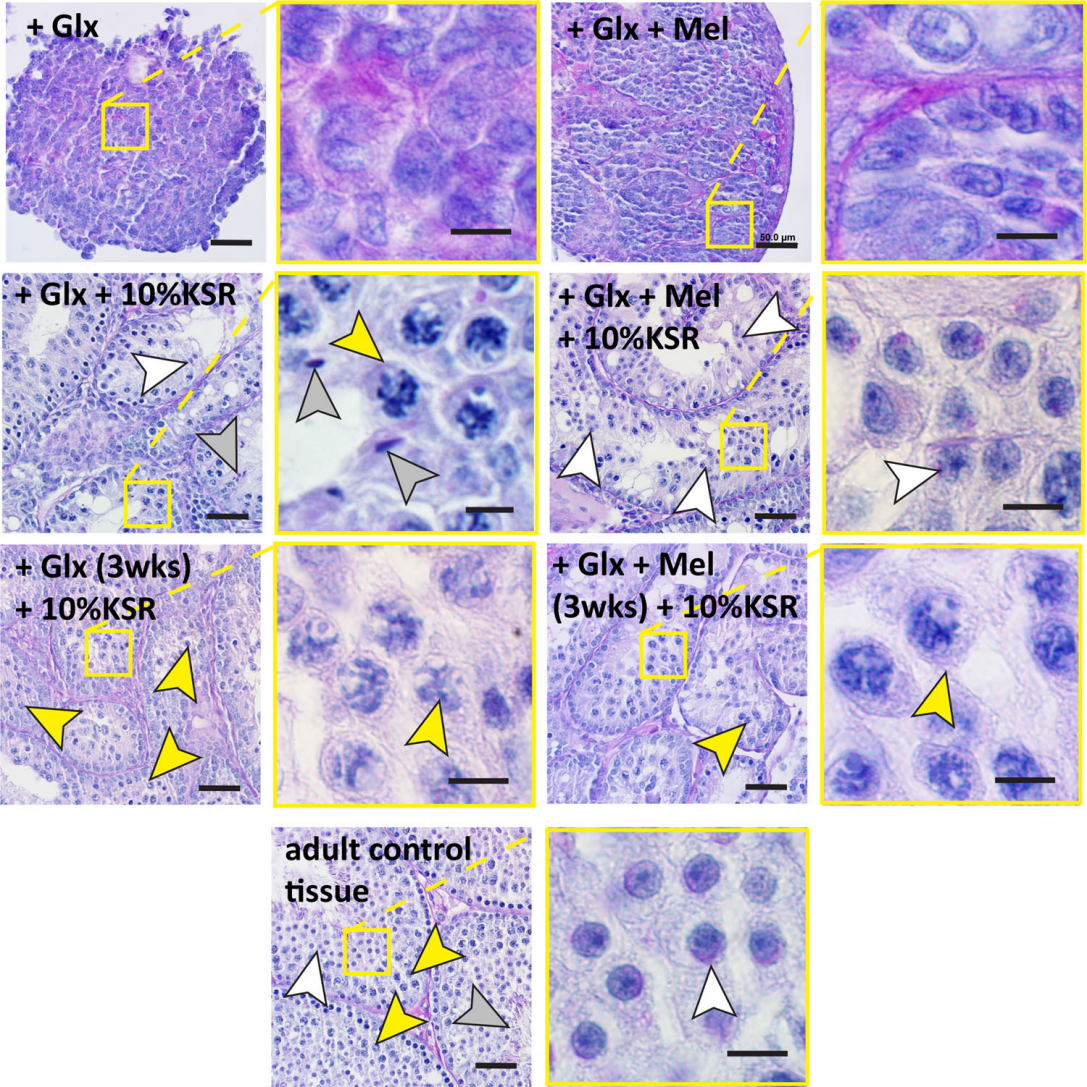
Morphologic evaluation of the Periodic Acid-Schiff (PAS) stained sections of Bouin’s solution fixed samples. Evaluation of 3 d*pp* murine testicular tissue cultured for 35 days in minimum essential medium alpha (MEMα) without supplementation with knockout serum replacement (KSR) (+Glutamax (Glx) or +Glx and melatonin (Mel)) did not show any differentiation of male germ cells into meiotic cells. The same type of tissue culture for 35 days with KSR supplementation (+Glx + 10% KSR or +Glx + Mel + 10% KSR) exhibited the presence of post-meiotic cells (round and elongated spermatids: white and grey arrowheads, respectively). The supplementation with KSR for 2 weeks after 3 weeks (weeks) of culture without KSR (+Glx (5 weeks) + 10% KSR (2 weeks) or +Glx + Mel (5 weeks) + 10% KSR (2 weeks)) resulted in the initiation of germ cell meiosis (yellow arrowheads). The obtained meiotic and post-meiotic cells exhibited specific features of meiotic (pachytene spermatocytes: yellow arrowheads) and post-meiotic cells (round and elongated spermatids: white and grey arrowheads, respectively) that were recognized in testicular tissue of adult mice control. Scale bars: 50 µm: overviews, 10 µm: higher magnifications.

Morphologic evaluation of the PAS staining showed that tissue fragments cultured in medium supplemented with Glutamax, melatonin, or a combination, as well as the un-supplemented culture medium, exhibited seminiferous tubules containing spermatocytes, round and elongated spermatids after 35 days of culture (Fig. 3). Spermatocytes and round spermatids were observed in all tissue fragments cultured with 10% KSR and melatonin, Glutamax or the combination of both. Elongated spermatids could be recognized in 33% of tissue fragments cultured with 10% KSR and Glutamax, in 83% of fragments cultured with 10% KSR and melatonin and 67% cultured with melatonin and Glutamax.

### Effect of KSR on yH2AX Expression in Diploid, Double-Diploid and Haploid Cells

In order to evaluate the efficiency of the organ culture system in IVM of murine germ cells, murine testicular tissue cultured for 35 days in MEMα + melatonin, with or without 10% KSR supplementation, was digested to produce single cell suspension. The percentage of haploid cells produced by the culture was assessed relative to the total number of testicular cells by flow cytometry. The results showed that the percentage of haploid cells (1C) was 2.3 ± 0.8 and 1.9 ± 0.6%, for the tissue cultured with and without 10% KSR respectively, as shown in Supplementary Fig. 2A. The percentage of cells positive for γH2AX (a protein expressed in a subset of the diploid spermatogonia, step 7–8 haploid spermatids and double-diploid spermatocytes^3^) was evaluated as well. Within the haploid cell population, this percentage was significantly higher in cells from the tissue cultured for 35 days in 10% KSR compared to the cells from the non-cultured control testicular tissue at 3 d*pp* of age (55.3 ± 72 and 0%, respectively), as shown in Supplementary Fig. 2B. Meanwhile, this percentage was not significantly different between the cells from the tissue cultured in 0% KSR (13.9 ± 15%) and the cells from the control testicular tissue at 3 d*pp* of age.

### Effect of Different Concentrations of KSR

**Figure 4:**
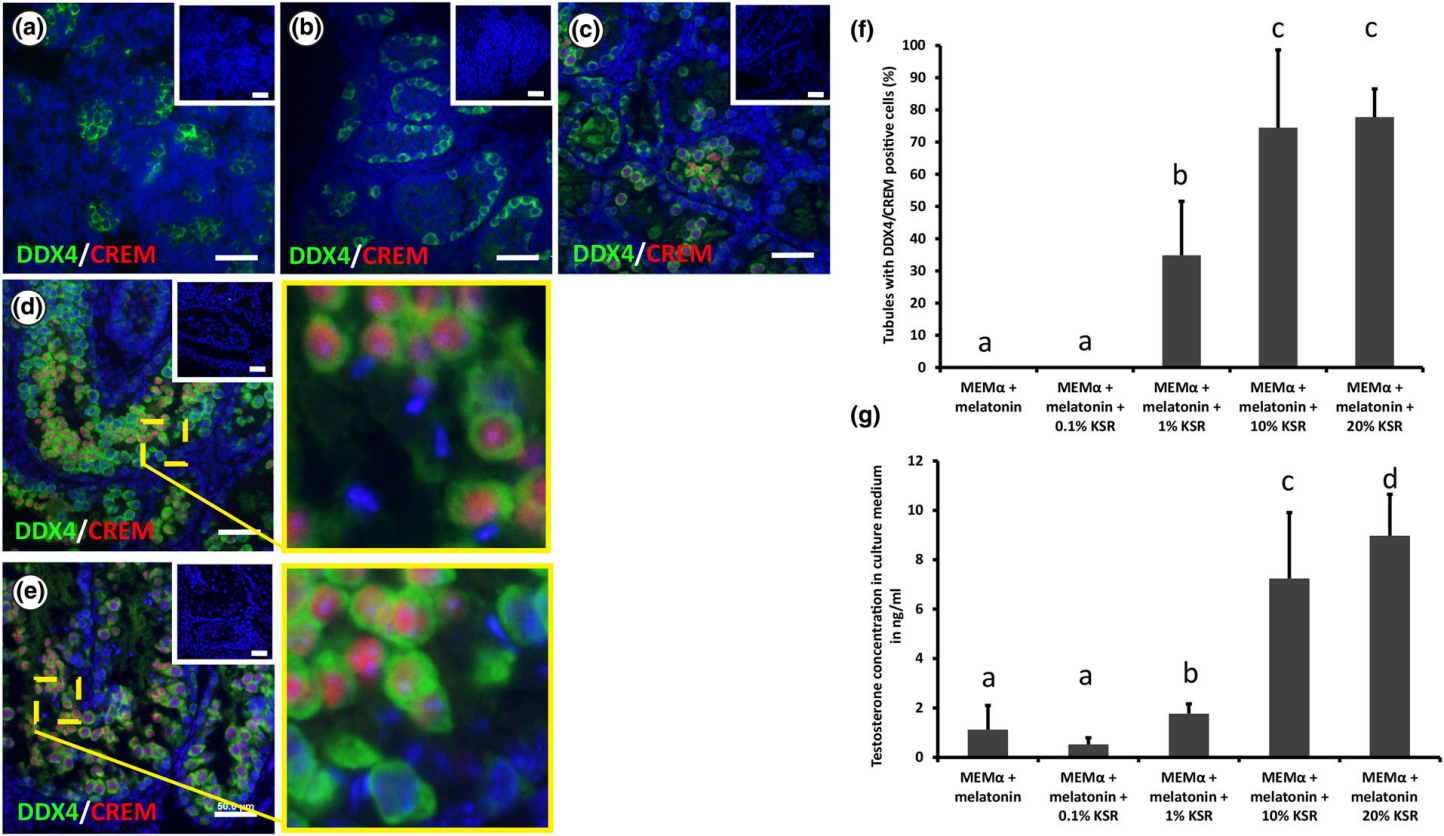
Effect of Knockout Serum Replacement (KSR) on murine pre-pubertal testicular tissue cultured *in vitro*. (a–e) Double immunofluorescence staining for DDX4 (green)/CREM (red) of paraformaldehyde (PFA) 4% fixed sections of 3 d*pp* mouse testicular tissue cultured for 35 days in Minimum Essential Medium alpha (MEMα) + melatonin (a), MEMα + 0.1% knockout serum replacement KSR + melatonin (b), MEMα + 1% KSR + melatonin (c), MEMα + 10% KSR + melatonin (d), and MEMα + 20% KSR + melatonin (e). Small pictures in the upper right corners represent the negative control. Scale bar is 50 µm. (f) Percentage of tubules containing DDX4/CREM positive cells after 35 days of culturing 3 d*pp* mouse testicular tissue in MEMα + melatonin, MEMα + 0.1% KSR + melatonin, MEMα + 1% KSR + melatonin, MEMα + 10% KSR + melatonin, or MEMα + 20% KSR + melatonin. Three biological replicates were evaluated for each time point. Values are represented in mean ± standard deviation. For statistical analysis, one-way ANOVA followed by Holm-Sidak post hoc analysis was performed. Different letters represent statistical significance (*p* < 0.001: a vs. c; *p* < 0.05: b vs. a and c). (g) Concentrations of testosterone in ng/ml in the culture medium after culturing 3 d*pp* mouse testicular tissue for 35 days in MEMα + melatonin, MEMα + 0.1% KSR + melatonin, MEMα + 1% KSR + melatonin, MEMα + 10% KSR + melatonin, or MEMα + 20% KSR + melatonin. Three biological replicates were evaluated for each time point. Values are represented in mean ± standard deviation. For statistical analysis, one-way ANOVA followed by Holm-Sidak post hoc analysis was performed. Different letters represent statistical significance. (*p* < 0.001: a vs. d; *p* < 0.01: a vs. c, b vs. c and d).

In order to investigate the effect of KSR on IVM of murine germ cells, different concentrations of KSR (0, 0.1, 1, 10, and 20%) were added to the culture medium and the histology was analyzed after 35 days of culture (Fig. 4).

The results showed that KSR has actually a prominent effect on spermatogenesis *in vitro*, in a dose dependent manner, which proves that KSR is essential for IVM of murine germ cells. Significantly more cells per tubule could be observed in conditions containing 10% KSR as supplement to the culture medium (Table 1). When the culture medium was not supplemented or supplemented with 0.1% of KSR, there were no tubules at all containing CREM positive cells, indicating that there was no germ cell maturation beyond the late meiotic/post-meiotic stage. Nevertheless, supplementation with 1% KSR resulted in significant increase in the CREM index (34 ± 16%) compared to 0 or 0.1% KSR. However, no significant differences could be observed when comparing the numbers of DDX4 or DDX4/CREM expressing cells per tubules in those conditions (Table 1).

The supplementation with 10 and 20% of KSR resulted in a significant increase in the CREM index (74 ± 24 and 77 ± 8% respectively) compared to no supplementation or supplementation with 0.1 or 1% of KSR. However, there was no such significant difference when supplementation with 10% was compared to supplementation with 20% of KSR. The same result could be observed, when evaluating the numbers of cells expressing DDX4 or DDX4/CREM, which showed in both conditions a significant increase compared to no supplementation or supplementation with 0.1 or 1% KSR (Table 1).

PAS staining showed that no pachytene spermatocytes could be observed when the culture medium contained no KSR or 0.1% KSR. Meanwhile, round and elongated spermatids could be observed only when the culture medium was supplemented with 10% KSR or 20% KSR (in 83 and 67% of analyzed tissue fragments, respectively) (Fig. 3). When tissue samples were cultured for 3 weeks without KSR and then for additional 2 weeks with 10% KSR, the initiation of germ cell differentiation and meiosis could be observed, regardless the presence of melatonin (Fig. 3). Few round spermatids could be observed in 33% of all tissue fragments analyzed. The evaluation of DDX4 and DDX4/CREM expressing cell numbers per tubule, revealed a significant higher number of DDX4/CREM positive cells in conditions with melatonin. However, the total number of DDX4 expressing cells was higher in the condition without melatonin (Table 1).

In order to investigate the effect of the different KSR concentration on the testosterone production by the testicular tissue cultured *in vitro*, the medium was collected every week and testosterone levels in the collected media were measured. The results were consistent with the CREM and morphologic evaluation results, where testosterone levels increased with increasing KSR concentration. Supplementing the culture medium with 10% or 20% KSR resulted significantly in the highest testosterone level after 5 weeks of culture (7 ± 2 and 8 ± 1 ng/mL respectively), compared to supplementation with 0.1 or 1% KSR or no supplementation (0.5 ± 0.2, 1 ± 0.3, and 1 ± 0.9 ng/mL respectively). However, there was no significant difference between the produced testosterone levels after supplementation with either 10 or 20% KSR (Fig. 4g). This pattern of testosterone production was persistent in all of the culture media collected every week, from the first week of culture till the end of the culture at the fifth week (Supplementary Fig. 3).

## Discussion

The idea behind the organ explant cultures is quite simple; placing the cultured tissue at the interface between the liquid medium and the gaseous phase maintains the cellular architecture and ensures access to both nutrients and oxygen simultaneously. In their study, Sato and colleagues used testicular tissue from genetically modified C57/BL6 mice which expressed green fluorescent protein under the promoters of the post-meiotic markers ACROSIN and GSG2.^23^ In the current study we employed wild-type C57/BL6 mice to confirm the results obtained before^4^ and to use the same genetic background for the murine tissue used by Sato *et al.* 23 As reported by other researchers, who found similar results using wild-type CD-1 mice,^2^,^7^ our study demonstrates that wild-type C57/BL6 mice can also be used for testicular fragment cultures. Testicular tissues from 3 d*pp* mice were used to ensure that the most advanced germ cells were undifferentiated spermatogonia.^11^

The results of the DDX4/KI-67 staining showed that the tissue cultured for 35 days had the greatest proliferation index compared to the tissue cultured for 18 days or 56 days. However, a decrease in numbers of DDX4 and DDX4/CREM positive cells could be already observed from day 35 and 56 in culture. This as well as the reduction of proliferation around 56 days of culture shows that the organ culture system might not be stable for germ cell differentiation beyond 35 days. This result confirms what was reported previously.^4^

Our explants were cultured routinely for 35 days, to match the time needed *in vivo* to show full spermatogenesis.^18^ MEMα culture medium was used as the basal medium, since it showed favorable outcomes previously.^23^,^24^ CREM was used as late/post meiotic marker in this study, as it is expressed in the mammalian male germ cell from the late pachytene stage till the mature spermatid.^8^

We have investigated here the effect of three supplements; Glutamax, melatonin, and KSR. Glutamax is a dipeptide of alanine and glutamine, which has less cytotoxic effects than glutamine when added to the cell culture.^5^ We have shown previously positive effects of Glutamax on rat gem cell viability *in vitro*.^19^ Hence, we hypothesized that it could have a positive impact on the cultured murine germ cells. Melatonin, on the other hand, has an anti-cytotoxic effect through scavenging the reactive oxygen species (ROS) as an antioxidant.^15^,^17^ Based on promising results for melatonin in previous work using testicular tissue of CD-1 mice^4^ and rats,^20^ we hypothesized its positive effect in our murine testicular organ cultures. The concentration of melatonin we used was 10^−7^ M, which was used previously to produce the antioxidant effect.^4^,^26^,^36^,^37^

The major modification that Sato *et al*. have introduced to the culture system was actually the substitution of fetal bovine serum (FBS) with KSR. When FBS was used earlier in similar attempts, it produced a meiotic arrest, where the germ cells could not pass through meiosis.^27^,^28^ Notably, KSR was intended to help in the propagation and to keep stem cells in a pluripotent state, preventing them from differentiation.^1^ Surprisingly, using 10% KSR resulted in completed meiosis, producing mature haploid germ cells. However, the optimal concentration of KSR was unreported when we began this work.

The results showed that no significant difference could be observed in the number of tubules with CREM positive or KI-67 positive cells after supplementation with melatonin, Glutamax, or a combination. However, we observed a significant**ly** higher number of cells per tubule expressing DDX4 and DDX4/CREM when melatonin was added as supplement to the basic culture medium. In addition, we could see some elongated spermatids with melatonin supplementation suggesting a supportive effect on germ cell differentiation. Additional experiments need to be done on the role of melatonin in a dose dependent manner.

In short-term cultures (1–7 days) of seminiferous tubules at defined stages of the epithelial cycle, spermatogenesis proceeded normally through meiotic divisions, and added hormones (e.g., insulin, FSH, testosterone and retinoic acid) had not much effect on the efficiency.^30^,^32^,^33^ However, in the tubule cultures spermiogenesis did not advance beyond elongation.^31^

When we supplemented the culture medium with different concentrations of KSR and compared them, we have seen a dose response pattern of germ cell differentiation in relation to the KSR concentration, shown by the CREM staining and the morphologic evaluation of the different developmental stages of the differentiated germ cell (spermatocytes, round spermatids, and elongated spermatids). Moreover, we could also see the same effect of KSR on the maturation of the tubules (e.g., formation of the lumen in the tubules; increase of total cell numbers per tubule). This pattern proves that by increasing the concentration of KSR from 0 to 10% we get an improvement in the germ cell differentiation. Nevertheless, when we go beyond the 10% (to 20% KSR), there was no added benefit on the germ cell differentiation that could be observed, based on the CREM staining results, proving that the 10% KSR concentration is the optimal concentration.

The same pattern was seen when we investigated the levels of testosterone production by the testicular tissue in response to the different KSR concentrations, which might explain the effect on germ cell differentiation. As the KSR concentration increased from 0 to 10%, the testosterone levels increased as well. Meanwhile, when the concentration was further increased to 20%, there was no significant difference in testosterone levels, which matches with the germ cell differentiation results. Testosterone is essential in spermatogenesis by its effect on Sertoli cells and peritubular myoid cells (PMCs).^35^ Hence, optimal testosterone concentrations are needed in order to accomplish spermatogenesis. Since KSR contains mainly lipid-rich albumin,^13^ it could be that the lipids in the KSR are responsible for the increase in testosterone production.

Another effect of the KSR could be observed when we did quantitative analysis of testicular cell populations by flow cytometry. The results revealed that the 10% KSR resulted in a significantly higher 1C cells in the total population, when compared to the 3 d*pp* control. This result confirms the presence of post-meiotic mature germ cells in the cultured tissue. Moreover, the yH2AX expression results showed that the expression was significantly higher in the cells from the tissue cultured with 10% KSR compared to the control cells from 3 d*pp* testicular tissue, in both 1C and 2C cells. The yH2AX is expressed in the mouse male germ cell in a subset of the diploid spermatogonia and stage 7–8 haploid spermatids, just before the onset of spermatid elongation.^3^ Therefore, these results further confirm presence of the haploid spermatids in the culture.

Interestingly, the progression of differentiation of murine germ cells was similar in our *in vitro* conditions with at least 10% KSR compared to the situation found *in vivo*. However, future studies on stage specific expression patterns are needed to compare this aspect of spermatogenesis *in vitro* vs. *in vivo*, in detail.

In conclusion, we report here that the duration of culture of murine testicular explant culture had an impact on the germ cell proliferation and on the efficiency of germ cell maturation up to the late/post meiotic stage. Moreover, supplementation with melatonin and Glutamax showed a positive effect on germ cell differentiation efficiency. In addition, we could observe some elongated spermatids only with melatonin supplementation. The results revealed also that KSR supplementation had a prominent effect on tubule maturation, germ cell maturation and testosterone production, with a concentration of at least 10%.

## Electronic supplementary material

Below is the link to the electronic supplementary material.

Supplementary material 1 (TIFF 6422 kb)

Supplementary material 2 (TIFF 240 kb)

Supplementary material 3 (TIFF 226 kb)

Supplementary material 4 (PDF 476 kb)
