# Supplementary figures and images for: Knock-Out Serum Replacement and Melatonin Effects on Germ Cell Differentiation in Murine Testicular Explant Cultures
Source: Ann Biomed Eng. 2017 May 9;45(7):1783–94. doi: 10.1007/s10439-017-1847-z (PMC5489632; doi:10.1007/s10439-017-1847-z)

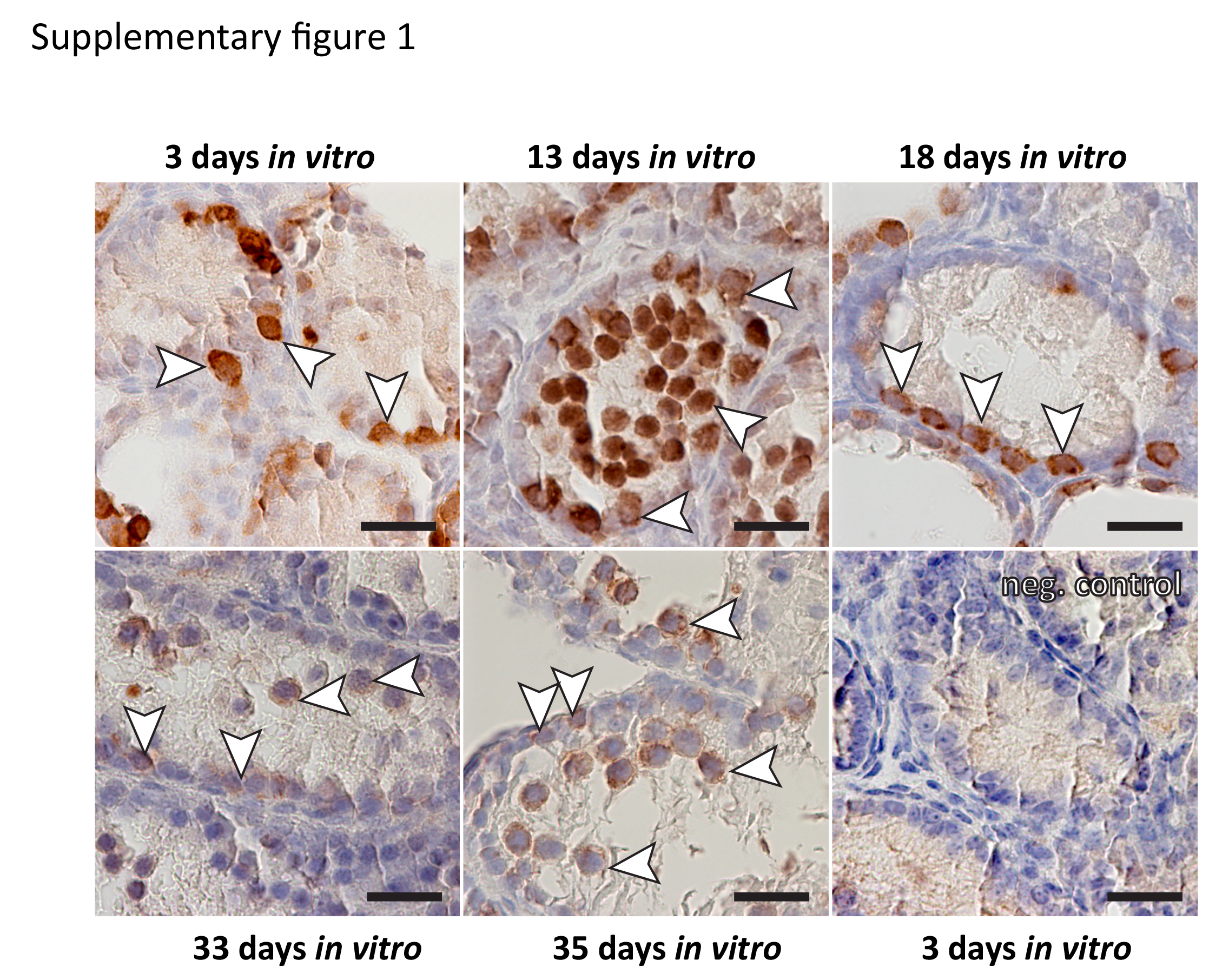

Supplement: Supplementary file 1 — Supplementary material 1 (TIFF 6422 kb) [file 10439_2017_1847_MOESM1_ESM.tif]

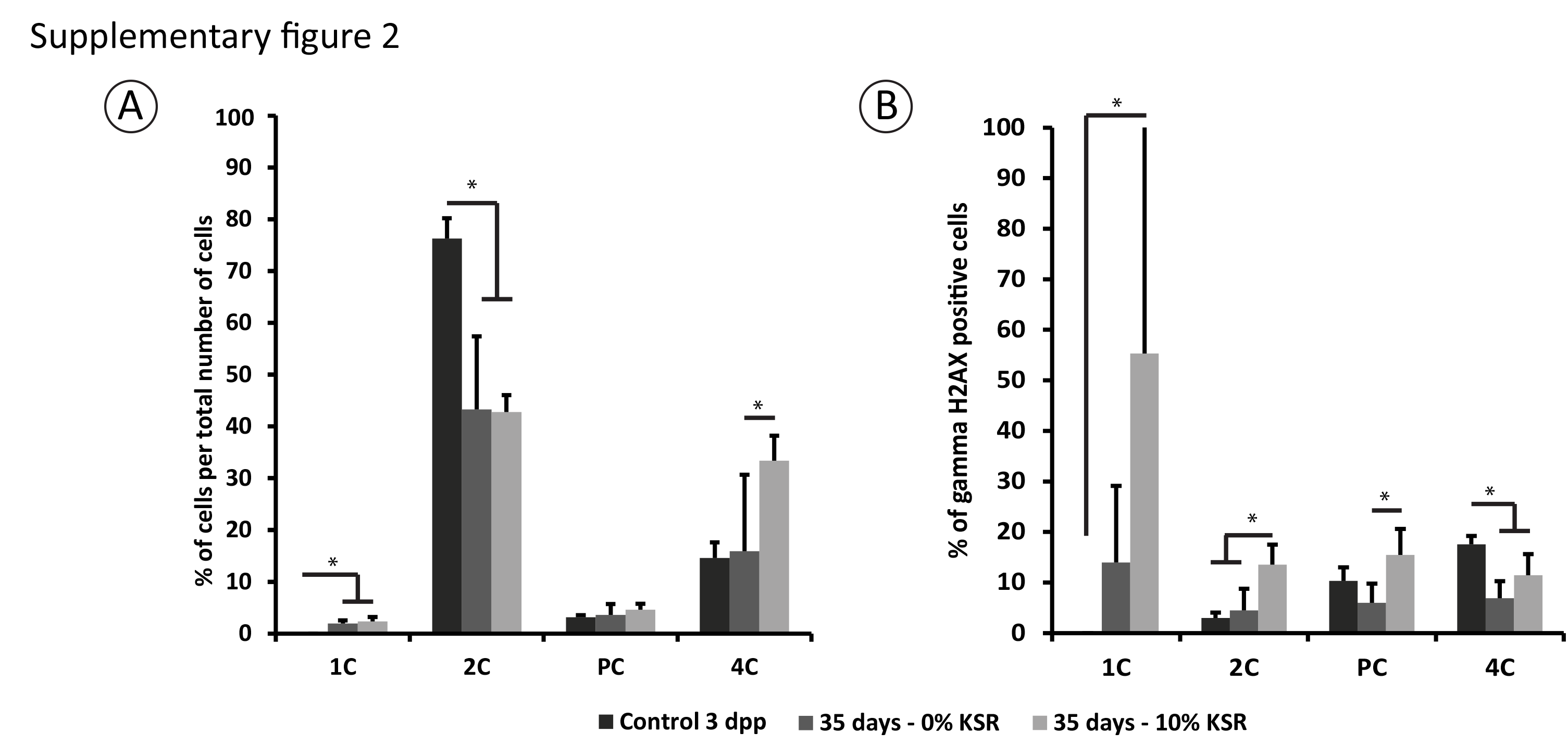

Supplement: Supplementary file 2 — Supplementary material 2 (TIFF 240 kb) [file 10439_2017_1847_MOESM2_ESM.tif]

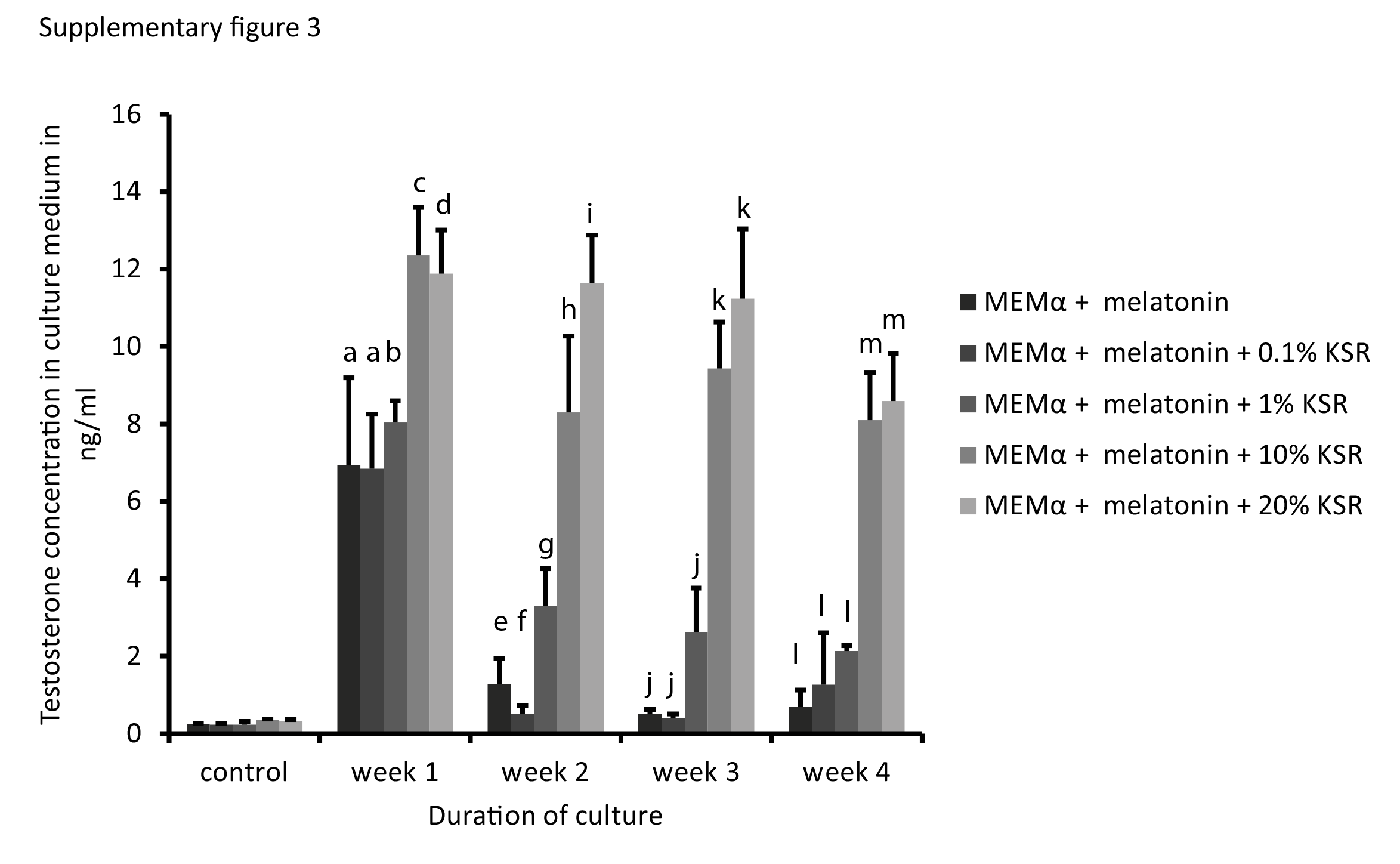

Supplement: Supplementary file 3 — Supplementary material 3 (TIFF 226 kb) [file 10439_2017_1847_MOESM3_ESM.tif]
