# Supplementary material for: Knock-Out Serum Replacement and Melatonin Effects on Germ Cell Differentiation in Murine Testicular Explant Cultures
Source: Ann Biomed Eng. 2017 May 9;45(7):1783–94. doi: 10.1007/s10439-017-1847-z (PMC5489632; doi:10.1007/s10439-017-1847-z)
Supplement: Supplementary file 4 — Supplementary material 4 (PDF 476 kb) [file 10439_2017_1847_MOESM4_ESM.pdf]

Title: Knock-out serum replacement and melatonin effects on germ cell differentiation in murine testicular explant cultures

Authors: Ahmed Reda[a], Halima Albalushi[a], Sheyla Cisneros Montalvo[b], Mirja Nurmio[b], Zeliha Sahin[c], Mi Hou[a], Niels Geijsen[c], Jorma Toppari[b], Olle Söder[a], Jan-Bernd Stukenborg[a,*]

[a]Department of Women's and Children's Health, Pediatric Endocrinology Unit; Q2:08; Karolinska Institutet and Karolinska University Hospital, SE-17176 Stockholm, Sweden. [b] Department of Physiology, Institute of Biomedicine, University of Turku, and Department of Pediatrics, Turku University Hospital, Turku, Finland, [c]Hubrecht Institute–KNAW and University Medical Center Utrecht, Utrecht, The Netherlands.

Supplementary Figure legends

Supplementary Figure 1:

DAZL immunohistochemistry staining for paraformaldehyde (PFA) 4% fixed sections of 3 d*pp* mouse testicular tissue cultured in minimum essential medium alpha (MEMα) + 10% knockout serum replacement (KSR) for 3, 13, 18, 33, and 35 days in vitro. White arrowheads indicate the positive cells. Picture in the lower right corner is for the negative control. Scale bar is 50µm.

Supplementary Figure 2:

Quantitative analysis of testicular cell populations by flow cytometry. (A) Percentage of cells per total number of cells showing 1C, 2C, PC, or 4C **(cell nuclei were stained with FxCycle Far Red Stain)** after flow cytometry of 3 d*pp* mouse testicular cells as a control or after flow cytometry of 3 d*pp* mouse testicular tissues cultured for 35 days in Minimum essential medium alpha (MEMα) + melatonin or MEMα + 10% knockout serum replacement (KSR) + melatonin. **Four biological replicates were evaluated for each condition.** Values are represented in means ± standard deviation. For statistical analysis, ANOVA on ranks followed by Tukey's method for post-hoc analysis (for 2C) and one-way ANOVA followed by Holm-Sidak post-hoc analysis (for 1C, PC, and 4C) were performed. *: $P < 0.05$. (B) Percentage of γH2AX positive cells **within each of the** 1C, 2C, PC, or 4C cell **population (all cell nuclei were stained with FxCycle Far Red Stain)** after flow cytometry of 3 d*pp* mouse testicular cells as a control or after flow cytometry of 3 d*pp* mouse testicular tissues cultured for 35 days in MEMα + melatonin or in MEMα + 10% KSR + melatonin. **Four biological replicates were evaluated for each condition.** Values are represented in means ± standard deviation. For statistical analysis, ANOVA on ranks followed by Tukey's method for post-hoc analysis (for 1C) and one-way ANOVA followed by Holm-Sidak post-hoc analysis (for 2C, PC, and 4C) were performed. *: $p < 0.05$. C refers to the DNA contents, and PC stands for proliferating cells (S-phase).

Supplementary Figure 3:

Concentrations of testosterone in ng/ml in the culture medium after culturing 3 d*pp* murine testicular tissue for 1, 2, 3, and 4 weeks in minimum essential medium alpha (MEMα) + melatonin, MEMα + 0.1% knockout serum replacement (KSR) + melatonin, MEMα + 1% KSR + melatonin, MEMα + 10% KSR + melatonin, or MEMα + 20% KSR + melatonin. **Three biological replicates were evaluated for each condition and each time point.** Values are represented in means ± standard deviation. For statistical analysis, one-way ANOVA followed by Holm-Sidak post-hoc analysis was performed. Different letters represent statistical significance (p< 0.001: i vs e, f, and g; h vs e and f; j vs k; l vs m; p< 0.01: a vs c, g vs h; p <0.05: a vs d; b vs c and d; f vs g; h vs i).

Title: Knock-out serum replacement and melatonin effects on germ cell differentiation in murine testicular explant cultures

Authors: Ahmed Reda[a], Halima Albalushi[a], Sheyla Cisneros Montalvo[b], Mirja Nurmio[b], Zeliha Sahin[c], Mi Hou[a], Niels Geijsen[c], Jorma Toppari[b], Olle Söder[a], Jan-Bernd Stukenborg[a,*]

[a]Department of Women's and Children's Health, Pediatric Endocrinology Unit; Q2:08; Karolinska Institutet and Karolinska University Hospital, SE-17176 Stockholm, Sweden. [b] Department of Physiology, Institute of Biomedicine, University of Turku, and Department of Pediatrics, Turku University Hospital, Turku, Finland, [c]Hubrecht Institute–KNAW and University Medical Center Utrecht, Utrecht, The Netherlands.

Supplementary material and methods:

Animals

Wild-type C57/BL6 pregnant mice were purchased from Charles River (Sulzfeld, Germany), transported with their mothers to Karolinska Institutet (Stockholm, Sweden). Pregnant C57/Bl6 mice were housed at the Animal Center of the University of Turku (Turku, Finland) in a controlled environment (12 h light/12 h darkness; temperature 21±1°C) with free access to food and water *ad libitum*. The day of birth was designed as day 0. Male pups were sacrificed on postnatal day 3 by cervical dislocation or decapitation and testes were collected for *in vitro* cultures.

Testicular tissue culture

The testicular explant culture methods for testicular tissue, described originally by Sato *et al.* 2011 [26], was used to culture the mouse pre-pubertal testicular tissue. To prepare the agarose pillar, pre-autoclaved 0.7% SeaKem® LE agarose (50004, Lonza, Basel, Switzerland) was mixed with the relevant culture medium 1:1 v/v to give a final concentration of 0.35% agarose. The agarose-mixture medium was placed in 24 well plates, 1ml per well, and left to solidify at room temperature (RT). Afterwards, the solidified mixture was cut into small pillars (approximately 7mm diameter and 5 mm height) and placed in 6 well plates, 3 pillars per well. The relevant medium (2ml) was placed in each well of the 6 well plates, so that they reach the edge of the pillars without covering them. Testes from C57/Bl6 mice at the age of 3 days postpartum (d*pp*) were obtained after decapitation in Minimum Essential Medium alpha (MEMα, 22561-021, Gibco, Thermo Fisher Scientific, MA, USA) culture medium, supplemented with 1% penicillin/streptomycin (Pen/Strep, 15140-122, Gibco). Then, testes were minced into small pieces (2-3 pieces per single testis; ≈1mm^3 in size for each) using sterile scissors and forceps. The small pieces were placed on top of the agarose pillars, one piece per pillar, without being covered by culture medium. The basic culture medium consisted of (depending on the intended culture condition) either MEMα without Glutamax (22561-021, Gibco) or MEMα with Glutamax (32561-029, Gibco), both supplemented with 1% Pen/Strep. Moreover, two more supplements were tested in the culture medium in order to investigate different culture conditions; Knockout Serum Replacement (KSR, 10828-028, Gibco) and melatonin (M5250, Sigma Aldrich, Munich, Germany, final concentration 10^{-7} M). To prepare a stock solution, melatonin was dissolved in Dimethyl Sulfoxide (DMSO; D2650, Sigma Aldrich) to a concentration of 10^{-2} M and kept light-protected at -20°C. The working solution for melatonin was prepared by diluting the stock solution with MEMα

culture medium to a concentration of 10^{-4} M, followed by diluting in the assigned culture medium for each condition in 1:1000 to form 10^{-7} M final concentration. The different culture conditions used were; (1) MEMα + melatonin, (2) MEMα + glutamax, (3) MEMα + 10% KSR, (4) MEMα + glutamax + melatonin, (5) MEMα + glutamax + 10% KSR, (6) MEMα + glutamax + 10% KSR + melatonin, (7) MEMα + melatonin + 0.1% KSR, (8) MEMα + melatonin + 1% KSR, (9) MEMα + melatonin + 10% KSR, and (10) MEMα + melatonin + 20% KSR The tissue was cultured at 34.5°C, **normal oxygen tension (21%)** and 5% CO_2, while the culture medium was replaced once per week. The explants were cultured for up to 35 days in all culture conditions, with the exception that the MEMα + 10% KSR medium was evaluated at different times: 18, 35, and 56 days.

Embedding and sectioning

At the end of the culture, explants were collected and fixed either in Bouin's solution (HT10132, Sigma-Aldrich) or in formaldehyde 4% in PBS (02176, Histolab, Gothenburg, Sweden) at 4°C overnight. This was followed by dehydration in ascending ethanol concentrations (30%, 50% and 70% ethanol), each for 24 hours at RT. Dehydration of the samples continued with 80%, 96% and 99.6% ethanol respectively, each for 6 hours at least at RT. Samples were then transferred in butyl acetate (45860, Sigma-Aldrich) overnight at RT. Embedding of the samples was performed afterwards in melted paraffin (ParaplastX-TRA®, P3808, Sigma-Aldrich) at 61°C overnight. Samples were then left to cool down till solidification of the paraffin, and they were cut into 5μm sections using a Biocut sectioning machine (Reichert-Jung, NY, USA). The sections were placed on microscope slides (10143352, Superfrost Plus, Thermo Scientific, MA, USA) and dried in an oven at 37°C overnight.

Periodic acid-Schiff (PAS) staining and morphologic evaluation

Sections of the samples were de-paraffinized in xylene and rehydrated in descending ethanol concentrations; 99.6%, 96% and 70%, each for 10 minutes at RT. The PAS kit (101646, Merck, Darmstadt, Germany) was applied to stain the sections in accordance with the manufacturer's protocol. The sections were then washed in distilled water twice for 5 minutes each. Afterwards, the sections were incubated with periodic acid for 5 minutes and washed under tap water for 3 minutes, followed by washing in distilled water twice for 5 minutes each. The sections were then incubated with Schiff's reagent for 15 minutes at RT and washed again under tap water for 3 minutes, followed by washing in distilled water twice for 5 minutes each. Afterwards, sections were counter-stained with hematoxylin (Mayer's Hemalaun solution, 1092491000, Merck) for 2 minutes followed by washing under tap water. The sections were then de-hydrated in ascending ethanol concentrations; 70%, 96%, and 99.6%, followed by xylene, each for 10 minutes at RT. To mount the sections, Entellan® new (1079610100, Merck) was applied on the sections.

For the morphologic evaluation, the different types of germ cells were identified based on the morphologic aspects that were described earlier [25]. Primary spermatocytes were recognized by the large size of cell and nucleus compared to the other germ cell types, and by the threads of the condensed chromatin. Round spermatids were recognized by the smaller size of cell and nucleus compared to the other germ cell types. Elongated spermatids were characterized by their elongated nuclear structure. Additionally, both, round and elongated spermatids were identified by the presence of the acrosomal cap.

Immunostaining

Sections of formaldehyde 4% fixed samples were de-paraffinized in xylene and then rehydrated in descending ethanol concentrations; 99.6%, 96% and 70%, each for 10 minutes at RT. After washing in Tris-buffered saline (TBS) for 5 minutes, antigen retrieval was performed on the samples using citrate buffer (pH 6.0) at 95°C for 30 minutes, followed by cooling down at RT for 30 minutes. Subsequently, the sections were used either in **DDX4/KI-67** or in **DDX4/CREM** double staining. In both cases, the same protocol we described previously has been used [22]. The sections were washed in TBS for 5 minutes after antigen retrieval and blocked for the endogenous peroxidase by H_2O_2 30% in methanol for 30 minutes at RT. After washing again with TBS, the sections were blocked for non-specific binding by the incubation for 30 minutes at RT with a blocking buffer, formed of normal chicken serum 20% (NChS, C5405, Sigma-Aldrich) and bovine serum albumin 5% (BSA, 001-000-162, Jackson Immuno Research, PA, USA) in 1x TBS (TBS/NChS/BSA). Afterwards, the sections were incubated with either rabbit polyclonal anti-DEAD Box Protein 4 primary antibody (**DDX4**; ab13840, Abcam, Cambridge, UK 1:500, final concentration 2µg/ml) or rabbit immunoglobulins G (IgGs; ab27478, Abcam, 1:100, final concentration 2µg/ml) as a negative control overnight at 4°C, both diluted in the blocking buffer (TBS/NChS/BSA). On the second day, the sections were washed with TBS for 3 times, 5 minutes each, and incubated for 30 minutes at RT with Horseradish peroxidase (HRP)-conjugated chicken anti-rabbit secondary antibody (SC-2963, Santa Cruz, 1:200, final concentration 25µg/ml) diluted in blocking buffer (TBS/NChS/BSA). After washing three times with TBS, the TSA™ Plus Fluorescein System (NEL741001KT, Perkin Elmer Life Sciences, Boston, USA) was applied in accordance with the manufacturer's protocol. In order to strip away the antibodies from the sections, antigen retrieval was repeated one more time. After cooling down, the excess

peroxidase enzyme was blocked by incubating the sections with H_2O_2 30% in TBS/Tween-0.1% for 30 minutes at RT. The sections were washed with TBS for three times and incubated with the same blocking buffer (TBS/NChS/BSA) for 30 minutes at RT. Afterwards, the sections were incubated with either rabbit monoclonal anti-**KI-67** primary antibody (ab16667, Abcam, 1:200, final concentration 5µg/ml), rabbit polyclonal anti-**CREM** primary antibody (SC-440, Santa Cruz, 1:400, final concentration 0.5µg/ml) or rabbit IgGs as a negative control (1:400 – 1:40, final concentration 0.5 - 5µg/ml) overnight at 4°C, all diluted in the blocking buffer. On the third day, the sections were washed with TBS three times, followed by incubation with HRP-conjugated chicken anti-rabbit secondary antibody (SC-2963, Santa Cruz, 1:200, final concentration 25µg/ml), diluted in the same blocking buffer, for 30 minutes at RT. The sections were then washed three times with TBS and the TSA™ Plus Cyanine 3 System (NEL744001KT, Perkin Elmer Life Sciences) was applied according to the manufacturer's protocol. After washing with TBS, the sections were mounted in VECTASHIELD mounting medium with 4', 6-Diamidino-2-Phenylindole (DAPI; H-1500, VECTOR, CA, USA).

Immunohistochemistry with the anti-**DAZL** antibody (rabbit polyclonal, ab-34139; Abcam) was performed as follows:, antigen retrieval for sections of formaldehyde 4% fixed samples after de-paraffinization was performed in 50 mM glycine (pH 3.5; ≥90 °C maintained for 10 min) and the primary antibody was applied at 1.0 µg/mL for overnight incubation at 4°C in 0.1% bovine serum albumin (BSA)/TBS. Negative control sections were incubated with 0.1% BSA/TBS lacking primary antibodies. Subsequent steps were performed at RT, with TBS washes (3 × 5 min) between incubations. Primary antibody binding was detected using Vectastain ABC kit Universal according to the manufacturer's instructions (PK-7200, Vector Laboratories). Antibody binding was

detected as a brown precipitate following development with ImmPact DAB (SK-4105, Vector Laboratories), with Harris Haematoxylin used as counterstain. Sections were mounted under glass coverslips in Depex (BDH Laboratories, Poole, UK). At least three independent samples were examined.

In order to investigate whether melatonin and Glutamax had an additive effect when combined together on the proliferation of the germ cells *in vitro*, the **DDX4/KI-67** staining was evaluated. For the evaluation of the **DDX4/KI-67** staining, the number of tubules containing **DDX4** positive/**KI-67** positive cells per the total number of tubules in a section was counted, as an indication of germ cell proliferation. This number was compared mainly in three culture conditions; MEMα + melatonin + 10% KSR, MEMα + glutamax + 10% KSR, and MEMα + glutamax + 10% KSR + melatonin. A total of up to 239 tubules were counted for each culture condition.

In order to evaluate the **DDX4/CREM** staining, the number of tubules containing **DDX4** positive/**CREM** positive cells per the total number of tubules in a section was counted, as a marker for post-meiotic germ cell differentiation. This number was compared in the different culture conditions. A total of up to 315 tubules were counted for each c**ulture condition. A total of up to 122 tubules showing DDX4 expressing cells were evaluated. For each culture condition, blind analysis of germ cell numbers (DDX4 positive) and post-meiotic germ cells (DDX4/ CREM positive) was performed in sections by an experienced examiner using a fluorescence microscope (Eclipse E800, Nikon, Tokyo, Japan). Only round or slightly oval tubular cross-sections were included in the analysis of cell numbers.**

Microscopy

Examination of the PAS and immunostained sections was performed using an Eclipse E800 microscope (Nikon, Tokyo, Japan), while the pictures were obtained with a 12.5 million-pixel cooled digital color camera system (Olympus DP70, Tokyo, Japan).

Quantitative analysis of testicular cell populations by flow cytometry

Four cultured testis pieces from each treatment were pooled and prepared for flow cytometric analysis as previously described by Rotgers and collagues [24]. Briefly, cultured testis pieces were cut using McPherson-Vannas scissors and enzymatically digested with 1 mg/mL collagenase/dispase (10269638001, Roche, Basel, Switzerland), 1 mg/mL hyaluronidase (H3506, Sigma–Aldrich), 1 mg/mL DNAse1 (DN-25, Sigma–Aldrich). Cell suspensions were filtered through 35µm pore size filters (352235, BD Falcon, Franklin Lakes, NJ, USA) and subsequently fixed and permeabilized using 4% paraformaldehyde and 90% methanol. To assess the different germ cell populations in the testicular pieces, immunolabeling with mouse anti- γH2AX-Ser139 antibody (05-636, EMD Millipore Billerica, MA, USA), which is a marker for meiosis, was performed. Cells were blocked with 5% normal donkey sera and incubated with anti-phospho-Histone H2A.X antibody at a dilution of 1:500 for 1 hour at RT and donkey anti-mouse 488 secondary antibody (A21202, Invitrogen, Thermo Fisher scientific) for 1 hour at 37°C. The cell nuclei were stained with FxCycle Far Red Stain (F10348, Invitrogen). A fixed volume of samples were analyzed using a BD LSRII (Becton Dickinson, Franklin Lakes, NJ, USA) equipped with a High Throughput Sampler (HTS) - 96 well plate format. For FxCycle detection, a 633-nm red laser was used for excitation and emission was collected with 660/20 nm band pass filter. A 488nm laser was used for excitation of anti- γH2AX-Ser139 signal and emission was collected with a 530/30-nm band pass

filter. Analyses were performed with the noncommercial Flowing Software ver. 2.5 (Mr. Perttu Terho; Turku Centre for Biotechnology, Finland; www.flowingsoftware.com) as previously described in detail [24].

Testosterone assay

In order to evaluate the effect of the different concentration of KSR on testosterone production by the murine testicular tissues cultured *in vitro*, the culture medium was collected every week for 5 weeks and kept at -20°C. For the evaluation of testosterone levels, the collected media was thawed and the Enzyme Linked Immunosorbent Assay (ELISA) kit (EIA-1559, DRG instruments, Marburg, Germany) with an intra-assay CV < 5% and inter-assay CV < 10% was used according to the manufacturer's protocol. The absorbance was read at 450nm using the microplate reader Fluostar Omega (BMG LabTech, Ortenberg, Germany), while the standards provided with the kit were used to generate a standard curve. Standards and samples were run in duplicates. Culture media were placed in empty wells and collected every week as controls. Notably, we assumed that the size of the explant was the same in each well.
